# Cementless total hip arthroplasty with extended sliding trochanteric osteotomy for high congenital hip dislocation: A retrospective study

**DOI:** 10.1097/MD.0000000000006581

**Published:** 2017-04-07

**Authors:** Zhengliang Luo, Min Chen, Fei Hu, Zhe Ni, Xiaofeng Ji, Xiaoqi Zhang, Peng Cheng, Xifu Shang

**Affiliations:** Department of Orthopaedics, Anhui Provincial Hospital Affiliated to Anhui Medical University, Hefei, Anhui Province, China.

**Keywords:** extended sliding trochanteric osteotomy, high congenital hip dislocation, total hip arthroplasty

## Abstract

Total hip arthroplasty (THA) for high congenital hip dislocation (CHD) is technically demanding. The purpose of this retrospective study was to evaluate the results of cementless THA combined with extended sliding trochanteric osteotomy. We also assessed whether chronic low back pain was relieved after surgery.

The study included 19 patients (23 hips) with high CHD treated with cementless THA using extended sliding trochanteric osteotomy technique. Clinical and radiographic outcomes were evaluated.

Harris Hip Score, WOMAC score, visual analog scale for low back pain and Trendelenburg sign were significantly improved (*P* < 0.01) compared with the preoperative. Average limb-length discrepancy in the 15 unilateral hips was reduced from 38.2 ± 7.9 mm to 6.7 ± 4.1 mm (*P* < 0.01). No dislocation, deep vein thrombosis, or infection occurred. Two patients (8.7%) developed sciatic nerve palsy. One (4.3%) developed symptomatic greater trochanteric bursitis. Two (8.7%) sustained proximal femur shaft fracture during implantation of the femoral component. All femoral components showed successful bony ingrowth at the final follow-up. No stem subsidence was detected. There was no acetabular loosening. Bony union of the reattached greater trochanter was obtained in all hips. Wire breakage occurred in 3 hips (13%).

Cementless THA with extended sliding trochanteric osteotomy may be appropriate options for patients with high CHD.

## 1 Introduction

Congenital hip disease, a common cause of secondary osteoarthritis of the hip in young adults, provides a large total hip arthroplasty (THA) caseload for the orthopedic surgeon.^[1]^ High congenital hip dislocation (CHD) is the most severe, complex congenital hip disease according to Hartofilakidis classification. It corresponds to the Crowe type IV hip, in which the femoral head has subluxated more than 100% out of the hypoplastic true acetabulum and may even articulate with a hollow in the iliac wing that resembles a false acetabulum.^[2,3]^ THA for high CHD is technically demanding because of associated anatomical abnormalities, including deficient acetabular bone stock, deformity of the proximal femoral head, leg-length discrepancy, and severe soft-tissue contractures.^[3–5]^ As a result, a relatively high incidence of postoperative complications has been reported, especially nerve palsy.^[6]^

Extended sliding trochanteric osteotomy, introduced by Younger et al,^[7]^ is based on preserving the musculo-osseous-muscular sleeve which includes the gluteus medius, gluteus minimus, greater trochanter, and vastus lateralis. It allows physiological reconstruction of the soft tissue envelope of the hip. The osteotomy offers excellent exposure of the hip on both the acetabular and femoral sides and optimizes hip biomechanics. However, most surgeons selectively use greater trochanter slide osteotomy for complex primary and revision THA or fixation of acetabular fractures.^[8,9]^ We have used extended sliding trochanteric osteotomy to address congenital hip disease.

The first goal of this retrospective study was to review clinical and radiological outcomes, focusing on the complications in consecutive patients with high CHD underwent cementless THA combined with extended sliding trochanteric osteotomy. The second goal was to assess whether chronic low back pain was relieved after THA in these patients.

## 2 Material and methods

### 2.1 Ethical considerations

The Ethics Committee of Affiliated Provincial Hospital of Anhui Medical University reviewed and approved the present retrospective study. Signed informed consent was provided by all participants.

### 2.2 Patient population

Between September 2006 and October 2014, 19 patients (23 hips) with high CHD underwent primary THA. Three males and 16 females with an average age of 41.2 (range 21–65) years at the time of operation were included in the study. Four female patients underwent 2-stage bilateral THA, with a mean interval between operations of 6 (range 3–11) months. The main indication for surgery was gradually increasing pain due to secondary osteoarthritis that was unresponsive to nonoperative management. Patients who had undergone previous pelvic or proximal femoral osteotomy were excluded. Patient characteristics are shown in Table 1.

**Table 1:**
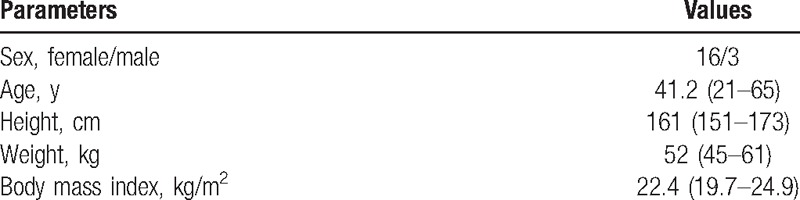
Demographics of the patients.

### 2.3 Surgical planning, technique, and postoperative procedures

Preoperative examinations were conducted in all patients to help with individual surgical plans. Preoperative anteroposterior radiography and computed tomography (CT) scans with 3-dimensional (3-D) reconstruction of the hip were used to confirm the dimensions of the true acetabulum, assess the proximal femoral deformities, and create a precise template of the acetabular and femoral components. A detailed surgical plan including the appropriate position, size, and anteversion angle of prosthetic components was then devised for reconstruction. The cementless hip prosthesis with ceramic on ceramic (LINK, Hamburg, Germany; Smith & Nephew, Tennessee) were used.

**Figure 1:**
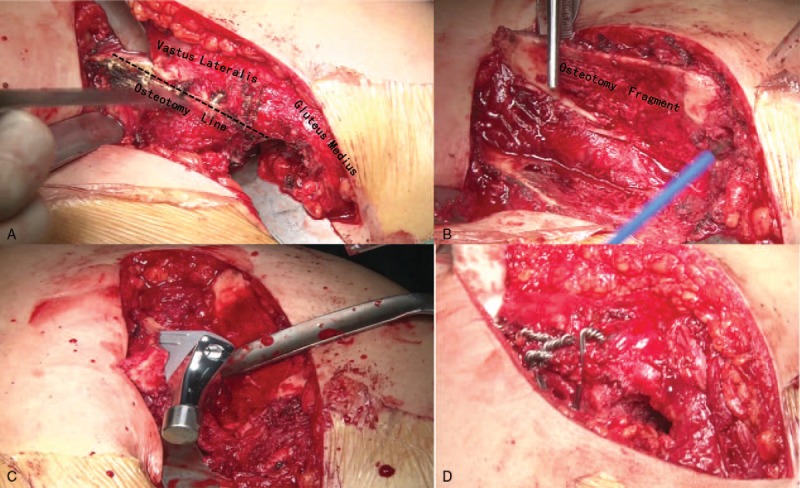
Position of osteotomy (A). The trochanteric fragment is shown (B). The stem is inserted into the proximal fragment of the femur (C), and trochanteric fragment is reattached to the lateral border of the femoral shaft (D).

All operations were performed with extended sliding trochanteric osteotomy (Fig. 1) by an experienced orthopedic surgeon (XFS). With the patient in the lateral decubitus position, the procedure was performed using a posterolateral approach in a laminar airflow operating room. After incising the skin and subcutaneous fascia, the gluteus maximus was bluntly separated in line with its muscle fibers. The piriformis and short external rotator muscles were palpated and detached with electrocautery at their insertions on the trochanter. They were then marked with sutures and lifted posteromedially. The extended sliding trochanteric osteotomy was made to preserve the continuity of the abductors (gluteus medius and minimus), the trochanter, and the vastus lateralis using an oscillating saw from posterior to anterior with hip joint slightly internal rotation. To achieve excellent exposure of the hip joint, the osteotomized greater trochanter fragment was then retracted anteriorly. The sciatic nerve was isolated and protected in all patients before the arthroplasty procedure. After careful and complete resection of the elongated hypertrophic capsule, fibrous scar, and osteophytes, we released the anterior rectus femoris, followed by insertion of the adductor, and iliopsoas. Lower limb traction was used to ensure that the femoral head achieved the level of true acetabulum without a need for further soft tissue release. With the hip in flexion, adduction, and internal rotation, the femoral neck was exposed, progressively osteotomized, and removed. We retained a certain length of the femoral calcar depending on the limb-length discrepancy.

After identifying the true acetabulum using the transverse acetabular ligament as the reference, we resected the fiber, fat tissue, and osteophytes to expose the entire rim of the acetabulum. The acetabulum was carefully reamed using the fossa as the reference to identify the anatomical center of the acetabulum until a hemispherical bony bed was developed. The acetabular component was then seated in the true acetabular position using the press-fit technique. When the defect of the acetabular wall was greater than 30%, a structural autogenous bone graft with two cancellous bone screws was applied to reconstruct the upper border of the acetabulum. The cavitary defects were filled with impacted cancellous morselized autogenous bone graft. Each acetabular cup was fixed with 2 or 3 dome screws.

After sequential reaming and rasping of the femoral canal, a trial implant was inserted into the proximal fragment of the femur, carefully controlling the anteversion of the stem. We prevented fracture of the proximal femur using prophylactic cerclage wires. A trial reduction of the hip was then performed to assess the stiffness of the circumarticular soft tissue and check its stability. If reduction was impossible, additional shortening was conducted by progressively resecting bone from the femoral neck. When the femoral component matched the broached intramedullary canal, it was inserted with a tight press fit.

During the reduction procedure, the sciatic nerve was palpated to ensure absence of any excessive tension. We protected it by extending the hip and flexing the knee at 90°, followed by gentle pressure on the femoral head plus prolonged traction. The limb was internally rotated to reduce the hip when the femoral head was on the edge of acetabulum. This position was maintained for a few days to readjust the tension of the sciatic nerve, which could obtain a minimal risk of injury of the sciatic nerve.

After the final reduction of the hip, the extended osteotomy fragment was reattached to the lateral border of the femoral shaft using several double-loop cerclage wires to maintain the abducted position of the hip with tension of the gluteus medius, which did not shorten the osteotomized fragment to any extent (Fig. 1). The wire knots were close to bone surface to prevent local problems such as pain or bursitis. Structured autogenous bone graft from the femoral head was placed at the reattachment site. The piriformis and short external rotator muscles were fixed to the reconstructed greater trochanter. A drainage tube was placed at articular cavity.

Prophylactic antibiotics were administered 1 hour before skin incision and continued for 72 hours postoperatively. Thromboembolic prophylaxis with subcutaneous enoxaparin or oral rivaroxaban was administered 8 hours after surgery and continued until hospital discharge. On the first postoperative day, isometric exercise was started with knee flexion on the bed. Patients were then allowed progressive flexion, extension, and abduction when there were no abnormalities of lower limb sensation or motion. Active abduction exercises were allowed until 6 to 8 weeks postoperatively. Weight bearing was progressively increased, depending on healing of the osteotomy. Patients walked with a toe-touch weight-bearing gait using 2 crutches, which were discarded only after radiography verified consolidation of the osteotomy site.

### 2.4 Clinical assessment and radiographic evaluation

Detailed clinical data were obtained for all patients preoperatively and postoperatively 1 month, and at the last follow-up. No patients were lost to follow-up. The data were reviewed by 2 independent authors. The perioperative blood loss was calculated as intraoperative blood loss plus postoperative blood loss. Intraoperative blood loss was estimated as the blood remaining in sponges and drapes and the volume in suction bottles. And the postoperative blood loss was measured by calculating the volume of blood in the drain bag. No attempt was made to estimate postoperative hematomas. The transfusion indications were that the patient had a reduction in hemoglobin exceeding 25% of the starting level and had clinical symptoms. Clinical outcomes were assessed using the Harris Hip Score (HHS),^[10]^ Western Ontario and McMaster Universities Osteoarthritis Index (WOMAC),^[11]^ and visual analog scale (VAS)^[12]^ for low back pain. The strength of the hip abductor muscles was assessed as having a positive or negative signs on the Trendelenburg test.^[13]^ The limb length of each patient was measured from the anterosuperior iliac spine to the mid-medial malleolus.

The preoperative, immediate postoperative, 1 year, and most recent anteroposterior radiographs of the pelvis were obtained for all patients. The results of fixation of the femoral prosthesis was evaluated as bony ingrowth, fibrous stable, or unstable according to the radiographic classification of Engh et al.^[14]^ Femoral component subsidence was defined as a change in its position greater than 5 mm by measuring the distance from the tip of the femoral component to a fixed point in the bone in the postoperative radiograph initially and at follow-up.^[15]^ For evaluating the state of the acetabular prosthesis, loosening was noted when a change in component position of either >3 mm or >3° according to Massin et al.^[16]^ Bone union of the reattached greater trochanter was classified as bone union, fibrous union, or nonunion according to the system developed by Hartofilakidis et al.^[17]^

### 2.5 Statistical analysis

All statistical analyses were performed using Statistical Package for Social Sciences version 13.0 for Windows (SPSS, Chicago, IL). Nonparametric Wilcoxon rank-sum tests were performed to evaluate differences in the HHS, WOMAC, and VAS. Chi-square test was used to evaluate differences in the Trendelenburg sign data. The *t* test was used to evaluate differences in the normally distributed limb-length discrepancy data. Statistical significance was set at *P* < 0.05, 2-tailed, for all analyses.

## 3 Results

All patients were followed up at an average of 5.7 years (range 1.2–9.3 years). The average operating time was 105 minutes (range 80–150 minutes). The average perioperative blood loss was 670 mL (range 450–1250 mL). The average perioperative allogenic blood transfusion was 4.3 U (range 2–6 U).

Clinical data are shown in Table 2. HHS, WOMAC score, VAS for low back pain and Trendelenburg sign and limb-length discrepancy were significantly differences compared with the preoperative (*P* < 0.01). Of the 23 hips, 19 (82.6%) were classified as good or excellent with HHS scores greater than 80.

**Table 2:**
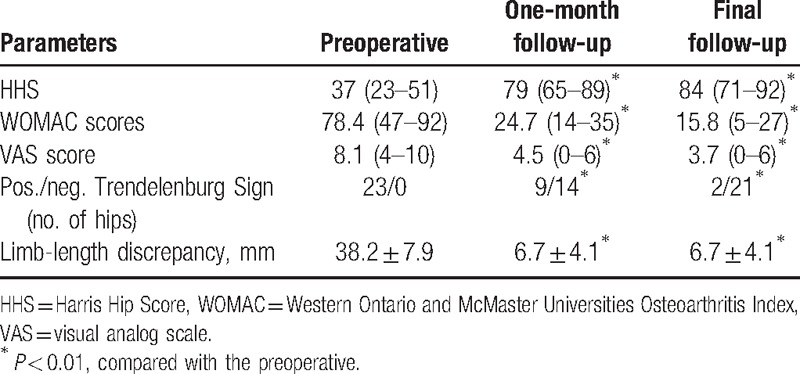
Clinical outcomes of the study.

No dislocation, deep vein thrombosis, or infection occurred in any of the patients. Two hips (8.7%) experienced sciatic nerve palsy on the first postoperative day. Both were fully recovered within 6 months with conservative treatment. One hip (4.3%) developed symptomatic bursitis of the greater trochanteric, which was treated conservatively with nonsteroidal antiinflammatory drugs (NSAIDs) and pain-free after treatment. Two hips (8.7%) sustained a proximal femur shaft fracture during component implantation. All fractures were nondisplaced and were stabilized with cerclage wires. Radiography indicated that they had healed 3 months later.

**Figure 2:**
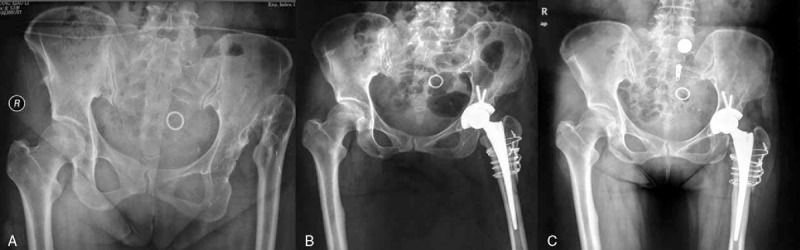
The preoperative (A), immediate postoperative (B), and 1-year (C) radiographic results in a 53-year-old woman with high congenital hip dislocation underwent a cementless total hip arthroplasty using treated with extended sliding trochanteric osteotomy.

All femoral components showed successful bony ingrowth, with no fibrous stable or unstable cases radiologically at the final evaluation. No stem subsidence was detected during follow-up. None of the acetabular components displayed loosening, and none required revision. Bony union of the reattached greater trochanter was obtained in all hips at 1 year after surgery (Fig. 2). Wire breakage was observed in 3 hips (13%).

## 4 Discussion

Patients with high CHD are often in severe pain and disabled. THA is an effective but challenging intervention to reduce pain and improve hip joint function. In this consecutive series, satisfactory results were achieved using cementless THA combined with extended sliding trochanteric osteotomy, progressive osteotomy of the femoral neck, a ceramic bearing surface, and proper soft tissue release. We believe that it is a safe technique for treating patients with high CHD. It offers better, safer hip exposure, and easier reconstruction of the distorted acetabular and femoral anatomy.

We placed the cementless acetabular components at the site of the true acetabulum for several reasons. First, this positioning is associated with a low rate of mechanical loosening because there is less shear force acting on the acetabular component here than at the site of a false acetabulum. A 15-year follow-up showed that the rate of loosening of the acetabular component placed at the true acetabulum site was 13% compared with 42% when it was placed proximal to the roof of the true acetabulum.^[18]^ Second, greater bone stock at the site of the true acetabulum provides better coverage than at the site of the false acetabulum. Additionally, a more stable acetabular component can be used.^[19]^ Third, this site restores normal hip biomechanics and abductor function, and improves the limb-length discrepancy.^[20]^

THA with cemented, cementless, or hybrid prostheses has been used to treat congenital hip disease.^[5,17,21]^ Survival analysis as the endpoint for revision has shown better outcomes for cementless prostheses. Perka et al^[22]^ reported a 97.5% survival rate for the acetabular component and 100% for the femoral stem after 9.3 years. However, polyethylene wear causing aseptic loosening was the major mechanism of prosthetic failure in their study. Another long-term follow-up study showed that the 14-year survivorship analysis of cementless THA with a third-generation ceramic-on-ceramic bearing surface was 97.9% for the cup, 97.8% for the stem, and 95.7% for the overall implants.^[23]^ In cases involving less than 70% coverage of the acetabular component, we preferred to use structural autogenous bone graft. We filled the cavitary defects with impacted cancellous morselized autogenous bone graft. In this series, we achieved good follow-up results, with no evidence of loosening of the acetabular components or need for revision.

There are several advantages of using the extended sliding trochanteric osteotomy, which compared with other subtrochanteric osteotomies performed with transverse, oblique, or double-chevron cuts.^[6]^ First, this osteotomy offers excellent exposure of the hip on both acetabular and femoral sides, reducing the need for aggressive retraction. It facilitates reduction of the hip and lowers the risk of nerve damage, for instance, to the sciatic or femoral nerve. Second, this osteotomy protects against greater trochanteric migration because it preserves the musculo-osseous-muscular sleeve hip abductors, maintaining the continuity of the greater trochanter and vastus lateralis. Extended sliding trochanteric osteotomy has been shown to increase abductor muscle length and abductor moment arm and to improve hip abductor biomechanics, resulting in stability of the hip and the osteotomized greater trochanter.^[24]^ We recommend beveling the osteotomized greater trochanter fragment (8–12 cm in length from the tip of the greater trochanter to the inferior border of the osteotomy, about half of the circumference of the proximal femoral diaphysis, and oblique to the inferior border) to obtain a broader osseous surface with stable double-loop cerclage wire fixation. The nonunion between the greater trochanter and the lateral border of the femoral shaft is a potential complication. Recent reports indicate that the rate of greater trochanteric nonunion with escape was 4.8% to 8.0%.^[25,26]^ In our series, all the osteotomy reattached sites in the patients healed within 1 year follow-up. We attribute this outcome to our operative technique.

Femoral abnormalities increase the risk of intraoperative femoral fracture ranging from 6% to 14% in previous series.^[4]^ Two proximal femur fractures occurred during implantation of the femoral component to obtain the maximum press-fit, even though prophylactic cerclage wires of the proximal femur shaft were used. The fractures healed radiologically at 3 months after surgery.

Clinical results have significantly improved, as shown by the HHS and WOMAC scores. It is important to note that the complication rate in this series was acceptable. The most common complication was wire breakage in 3 hips (13%) after union of the greater trochanter without migration. No painful outcomes or interference with the patients’ range of motion occurred. Another symptomatic complication was persistent pain caused by greater trochanteric bursitis, which occurred in 1 hip (4.3%) in our study, which was less than the reported prevalence of 14%.^[27]^ Wire knots were carefully twisted to lower local irritation. Fortunately, all patients were treated effectively with NSAIDs for pain unrelated to the wire breakage.

The Trendelenburg sign is a major assessment of the strength of hip abductor muscles. In this series, improvement of the Trendelenburg sign was statistically significant in 21 hips (91.3%). Similar to subtrochanteric osteotomy,^[28]^ postoperative limb-length discrepancy was also significantly decreased compared with the preoperative values. Edwards et al showed that the risk of nerve injury was apparently increased if preoperative limb-length discrepancy was more than 4 cm,^[29]^ suggesting an indication for subtrochanteric osteotomy. It had a prevalence of 3% to 15% in previous studies.^[30]^ Nine of our patients present preoperative limb-length discrepancy greater than 4 cm. However, 2 hips (8.7%) experienced sciatic nerve palsy and both of them completely recovered within 6 months after conservative treatment. We also recognized that it might increase the risk of sciatic nerve palsy if the surgical leg was extended greater than 4 cm. We felt the tension of sciatic nerve when hip reduction was performed with fully extended hip and flexed knee. Additional osteotomy would be carried out if the tension of sciatic nerve was too large.

We found that the patient's low back pain was significantly relieved after THA. Hip-spine syndrome, originally described by Offierski and MacNab more than 3 decades ago,^[31]^ is also associated with low back pain. The affected sagittal lumbar spine and pelvic tilt compensated for the leg-length difference, resulting in difficulty maintaining proper balance and a staggered gait. This study thus demonstrated the clinical benefits of THA in relieving low back pain in patients with congenital hip disease owing to shortening limb-length discrepancy.

The study limitations relate to the retrospective design and the relatively small number of cases, which may influence the results of the surgical technique. Furthermore, the precise mechanism of low back pain relief after THA needs to be clarified.

## 5 Conclusions

Cementless THA with extended sliding trochanteric osteotomy may be appropriate options for patients with high CHD.

## Acknowledgment

The authors thank the patients who participated and the staff involved in this study.
